# Metagenome Analysis Identified Novel Microbial Diversity of Sandy Soils Surrounded by Natural Lakes and Artificial Water Points in King Salman Bin Abdulaziz Royal Natural Reserve, Saudi Arabia

**DOI:** 10.3390/life14121692

**Published:** 2024-12-20

**Authors:** Yahya S. Al-Awthan, Rashid Mir, Fuad A. Alatawi, Abdulaziz S. Alatawi, Fahad M. Almutairi, Tamer Khafaga, Wael M. Shohdi, Amal M. Fakhry, Basmah M. Alharbi

**Affiliations:** 1 Department of Biology, Faculty of Science, University of Tabuk, Tabuk 71491, Saudi Arabia; falatawi@ut.edu.sa (F.A.A.); abalatawi@ut.edu.sa (A.S.A.); b.alharbi@ut.edu.sa (B.M.A.); 2 Biodiversity Genomics Unit, Faculty of Science, University of Tabuk, Tabuk 71491, Saudi Arabia; 3 Department of Medical Laboratory Technology, Prince Fahad Bin Sultan Chair for Biomedical Research, Faculty of Applied Medical Sciences, University of Tabuk, Tabuk 71491, Saudi Arabia; rashid@ut.edu.sa; 4 Department of Biochemistry, Faculty of Science, University of Tabuk, Tabuk 71491, Saudi Arabia; falrabae@ut.edu.sa; 5 King Salman Bin Abdulaziz Royal Natural Reserve Development Authority, Riyadh 12213, Saudi Arabia; t.khafaga@ksrnr.gov.sa (T.K.); w.elsheikh@ksrnr.gov.sa (W.M.S.); 6 Botany and Microbiology Department, Faculty of Science, Alexandria University, Alexandria 21568, Egypt; amalfakhry@live.com

**Keywords:** metagenome analysis, bacterial community, sandy soils, 16S rRNA gene, next-generation sequencing, natural reserve

## Abstract

Background: Soil microbes play a vital role in the ecosystem as they are able to carry out a number of vital tasks. Additionally, metagenomic studies offer valuable insights into the composition and functional potential of soil microbial communities. Furthermore, analyzing the obtained data can improve agricultural restoration practices and aid in developing more effective environmental management strategies. Methodology: In November 2023, sandy soil samples were collected from ten sites of different geographical areas surrounding natural lakes and artificial water points in the Tubaiq conservation area of King Salman Bin Abdulaziz Royal Natural Reserve (KSRNR), Saudi Arabia. In addition, genomic DNA was extracted from the collected soil samples, and 16S rRNA sequencing was conducted using high-throughput Illumina technology. Several computational analysis tools were used for gene prediction and taxonomic classification of the microbial groups. Results: In this study, sandy soil samples from the surroundings of natural and artificial water resources of two distinct natures were used. Based on 16S rRNA sequencing, a total of 24,563 OTUs were detected. The metagenomic information was then categorized into 446 orders, 1036 families, 4102 genera, 213 classes, and 181 phyla. Moreover, the phylum *Pseudomonadota* was the most dominant microbial community across all samples, representing an average relative abundance of 34%. In addition, *Actinomycetes* was the most abundant class (26%). The analysis of clustered proteins assigned to COG categories provides a detailed understanding of the functional capabilities and adaptation of microbial communities in soil samples. Amino acid metabolism and transport were the most abundant categories in the soil environment. Conclusions: Metagenome analysis of sandy soils surrounding natural lakes and artificial water points in the Tubaiq conservation area of KSRNR (Saudi Arabia) has unveils rich microbial activity, highlighting the complex interactions and ecological roles of microbial communities in these environments.

## 1. Introduction

Soil microbiomes are crucial for life’s sustainability as they carry out several vital tasks for ecosystems [1] (Garg et al., 2024). Moreover, these microbial communities play a significant role as they regulate soil health and functioning in biogeochemical cycles on Earth [1] (Garg et al., 2024). For instance, soil fertility, plant growth, nutrient cycling, and tolerance to abiotic and biotic stress are all significantly influenced by the soil microbiome [2,3] (Trivedi et al., 2021; Santos and Olivares, 2021). They exert control over soil structure through pore connectivity and aggregation, thereby facilitating the regulation of water flow and the circulation of nutrients and oxygen within the system [4] (Hartmann and Six, 2023). Moreover, they aid in nutrient cycling, bioremediation, and the production of antibiotics, enzymes, biofuels, and bioplastics [5] (Smith and Brown, 2023). It is essential to maintain a diverse soil microbiome for ecological restoration and agricultural productivity. Although there are numerous metabolically active microbes in their native environments, more than 99% of soil bacteria remain unculturable, which poses significant limitations on culture-dependent methods [6] (Chaudhary et al., 2019). Moreover, understanding the interaction between microbial communities and their metabolism is essential for their proliferation [7] (Kodera et al., 2022). Unlocking the soil microbiome presents new opportunities to discover hidden microorganisms and their potential applications [8] (Alidoosti et al., 2024). Metagenomics, a culture-independent technique based on omics, offers insights into environmental microbes that were previously uncultured [9] (Liu et al., 2022). By utilizing metagenomic studies and analyzing 16S rRNA gene sequences, it is possible to interpret and analyze the majority of previously inaccessible microbes [10] (Wilson and Piel, 2013).

The Tabuk region, located in the northwest of Saudi Arabia, contains a huge arid area with different ecosystems and distinctive habitats. This region is characterized by an arid climate, with temperatures ranging from 25 to 40 °C in the summer and 5 to 25 °C in the winter [11] (Al-Mutairi, 2022). Moreover, the Tabuk region’s various terrains and climatic conditions result in multiple terrestrial ecosystems with remarkable biological activity [12] (Ansari et al., 2022). Therefore, monitoring and assessing soil microbiomes in the Tabuk region is needed to understand the biodiversity of the sandy soils surrounding the water resources of natural lakes and artificial water points.

In this study, sandy soil samples surrounding the water resources of natural lakes and artificial water points in the Tubaiq conservation area of King Salman Bin Abdulaziz Royal Natural Reserve, which is the largest protected area in the Middle East [13] (Shabana et al., 2023), were collected in November 2023. Metagenomic sequencing analysis was performed to study the diversity of the microbial communities in sandy soil samples from natural and artificial distinct lacustrine environments. This study provides insights into types of bacteria and their distribution in relation to the actions of humans.

## 2. Methods

### 2.1. Study Location and Sampling

**Figure 1:**
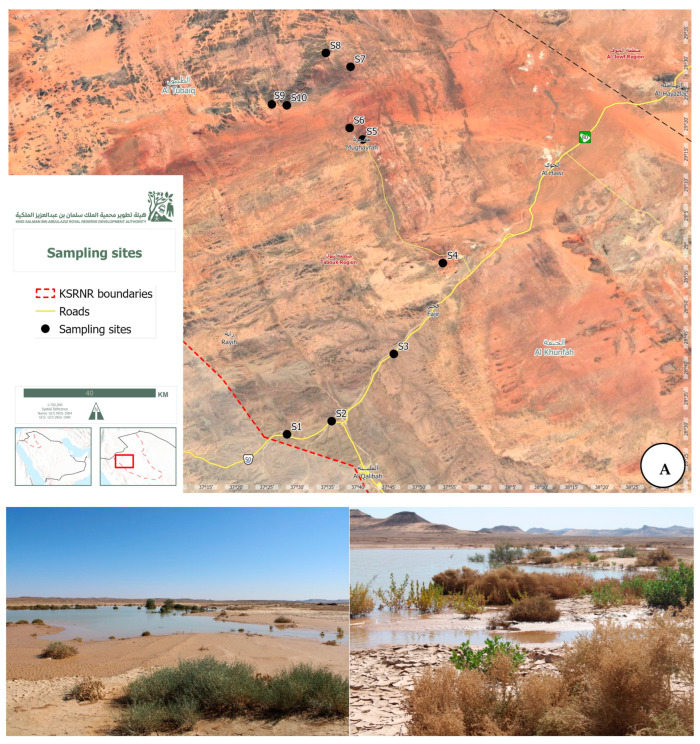

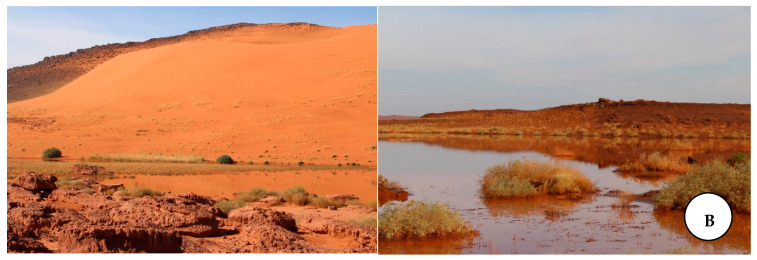
(**A**) A map showing research area inside within Al-Tubaiq region of the KSRNR. (**B**) Locations of the sampling sites within the Tubaiq area of KSRNR.

Samples of soil were taken from ten different places within the KSRNR, Saudi Arabia, in November 2023. The sites at the reserve’s edge, specifically S1 (28°29′06.7″ N 37°28′19.0″ E), S2 (28°31′14.4″ N 37°35′38.9″ E), and S3 (28°42′15.3″ N 37°45′50.1″ E), showed signs of human impact and had sparse vegetation. In contrast, sites S4 (28°57′09.6″ N 37°53′53.7″ E), S5 (29°17′27.4″ N 37°40′43.4″ E), S6 (29°19′20.5″ N 37°38′34.3″ E), and S7 (29°29′21.3″ N 37°38′44.5″ E), located in the reserve’s center, exhibited minimal human activity and featured small grasses. S9 (29°23′11.8″ N 37°25′50.1″ E), S10 (29°23′02.2″ N 37°28′16.1″ E), and S8 (29°31′37.4″ N 37°34′40.9″ E) were all at manmade water points that were remote from populated areas and devoid of vegetation. Figure 1 displays the research area’s location on a map. Three duplicate soil samples were gathered from each site. The samples were transported on ice to the University of Tabuk, Saudi Arabia’s Faculty of Science’s Biodiversity Genomics Unit. After being stored at −80 °C for DNA extraction and quantification, the samples were sent to the Genome Life Science Company in India for metagenomics and DNA sequencing.

### 2.2. Isolation and Quantitative Analysis of DNA

As directed by the manufacturer, DNA was extracted from the soil samples using an Alexgen Soil DNA Extraction Kit (CAT No. AG-SD50). A Qubit^®^ 4.0 fluorometer was then used to quantify the isolated DNA. The universal primers 8F and 1492R were used to amplify the 16S rRNA gene, and 1.8% (*w*/*v*) agarose gel electrophoresis was used to visualize the resultant amplicons.

### 2.3. Setting up the Library

Following the manufacturer’s guidelines, libraries for paired-end sequencing were constructed utilizing a DNA Library Kit from Twist Bioscience for Illumina^®^ (CAT No./ID 104119). Initially, 50 ng of DNA was enzymatically sheared into smaller fragments, which were then prepared for adapter ligation through A-tailing and end repair. The ends of the DNA fragments were fitted with an Illumina-specific adapter to facilitate the binding of the sequencing primers, PCR amplification, and library formation. High-fidelity PCR amplification was performed using a HiFi-PCR MasterMix (Takara, Kusatsu, Japan) to maximize yield. The quantity and quality of the amplified libraries were evaluated using an Agilent TapeStation (4150) system (Agilent Technologies, Waldbronn, Germany) with High-Sensitivity (D1000) Screen-Tape^®^, following the manufacturer’s instructions.

### 2.4. Sequencing and Cluster Generation

After assessing the Qubit concentration and analyzing the Tape Station profile, libraries were uploaded to an Illumina-Nova-Seq (6000) platform (Illumina, San Diego, CA, USA) for cluster generation and sequencing. Paired-end sequencing was performed, allowing the fragment templates to be sequenced in both directions. The oligos of the complementary adapters on the paired-end flow cell were hybridized with the library molecules. Following reverse strand re-synthesis, the adapter design facilitated the selective cleavage of forward strands, enabling sequencing from the fragment’s other end.

### 2.5. Generation of Data

To isolate individual samples, raw sequence data produced by the NovaSeq 6000 platform (Illumina, San Diego, CA, USA) were demultiplexed. Quality filtering was used to exclude adapter sequences and low-quality reads of the dataset (QV < 20). MEGAHIT (version 1.2.9), a specialized metagenome assembler made to handle large and complicated metagenomic datasets, was then used to de novo assemble the trimmed reads using default parameters [14] (Li et al., 2015).

### 2.6. Gene Prediction

To analyze the genomic profile in the metagenome samples, gene prediction was carried out on the assembled scaffolds using Prodigal (version 2.6.3) [15] (Hyatt et al., 2010) in the metagenome gene prediction mode. The predicted gene sequences (longer than 500 bp) were used to make the next functional and taxonomic evaluations.

### 2.7. Taxonomic Annotation

Using Kaiju, a quick and accurate metagenome classifier [16], quality-trimmed reads were taxonomically classified onto the assembled contigs (Menzel et al., 2016). Utilizing the Burrows–Wheeler transform (BWT) technique, this program finds the maximum number of protein-level matches in a reference database that contains annotated protein-coding genes from a group of microbial genomes. BLAST against the NCBI-nt database produced the taxonomy annotation. With the optional addition of fungi and microbial eukaryotes, the default reference database used in this work was made up of whole genomes from NCBI-RefSeq and the microbial subset of the nonredundant NCBI protein database (nr).

After converting the metagenomic readings into each of the potential reading frames, Kaiju looks for the most precise matches (MEMs) between amino acid sequences and the designated protein database. The approach effectively finds these matches using a modified backward search within the Burrows–Wheeler transform. The taxonomic identification of the lowest common ancestor (LCA) in the taxonomic hierarchy is assigned by Kaiju to reads that show matches to multiple database sequences. In this work, Kaiju analysis was applied to sequences produced by Prodigal. The following settings were used while using the standalone version of Kaiju: sequence low-complexity filter—enabled; database—nr; greedy run mode; permitted mismatches—5; minimum match score—75; and minimum match length—11 amino acids.

### 2.8. Diversity Analysis

Kaiju classification, a metagenomic taxonomic profiling classification tool [16], provided operational taxonomic unit (OTU) abundance data that were used to estimate alpha diversity, a within-sample measure of species richness and evenness (Menzel et al., 2016). The estimation of alpha diversity was performed using the estimate-richness function in the R-package phyLoseq (Version 1.48.0) [17] (McMurdie and Holmes, 2013), using the OTU table as the input. Several diversity indices, including Chao1, ACE, Shannon, Simpson, Inverse Simpson, and Fisher, were estimated as a result of this investigation.

### 2.9. Statistical Analysis and Data Visualization

Statistical analyses were executed using R (version 4.3.2) [18] (R Core Team, 2021). Data visualization and manipulation were performed with the aid of the tidyverse package (version 2.0.0) [19] (Wickham et al., 2019), ggplot2 package (version 3.4.4) [20] (Wickham, 2016), corrplot R package (version 0.92) [21] (Wei and Simko, 2021), and complex heatmap package (version 2.18.0) [22] (Gu et al., 2016) within the R environment.

## 3. Results

### 3.1. Study Site Characteristics

The 10 sandy soil samples used in this investigation came from two different settings: manmade water points and natural lakes. The geographic coordinates of these water resources show that they were geographically dispersed. With median values of 28.8° and 37.7°, respectively, natural lakes ranged in latitude from 37.5° to 37.9° and longitude from 28.5° to 29.3°. Conversely, the concentration of artificial water points was higher, with median values of 29.4° and 37.5° for longitude ranges between 29.4° and 29.5° and latitude ranges between 37.4° and 37.6°. The two settings were also differentiated by elevation profiles: manmade water points were at higher heights, with a median elevation of 871 m and a range of 764 to 919 m, whereas natural lakes had a median elevation of 814 m, ranging from 756 to 917 m. Significant variations were observed in the climate parameters. The median humidity of manmade water points was 19.5% (range: 18% to 20%), whereas that of natural lakes was 27.5% (range: 19% to 32%). There were also differences in temperature, with manufactured water points displaying a higher median temperature of 32.5 °C (range: 32 °C to 34 °C) and natural lakes having a median temperature of 29.5 °C (range: 27 °C to 37 °C). An overview of the features of the study site is given in Table 1, noting that sample 7 was lost during the process of analysis, and thereby, the related data of this sample were omitted from all the results of the current study.

### 3.2. The Taxonomic Makeup of the Microbial Communities in Soil

Using metagenomic sequencing, 24,563 operational taxonomic units (OTUs) were found and assigned to 181 phyla, 213 classes, 446 orders, 1036 families, and 4102 genera. *Pseudomonadota*, at the phylum level, dominated the communities with a mean relative abundance of 34% (range: 21.2–44.8%). Other predominant phyla included *Actinomycetota* (30%, 18.3–49%), *Bacillota* (12%, 0.7–25.9%), *Bacteroidota* (2%, 0.3–10.9%), and *Ascomycota* (0.5%, 0.1–1.4%).

At the order level, the most frequent were *Enterobacterales* (19%, 10.9–25.1%), *Micrococcales* (13%, 3.6–24.9%), *Lactobacillales* (5%, 0–10.6%), *Bacillales* (5%, 0.6–7.4%), and *Hyphomicrobiales* (4%, 1–8.3%). The most abundant classes were *Actinomycetes* (26%, 18.1–40.1%), *Gammaproteobacteria* (24%, 12.5–32.2%), *Bacilli* (11%, 0.6–17.9%), *Alphaproteobacteria* (8%, 2.9–13.5%), and *Betaproteobacteria* (2%, 0.4–6.6%). Dominant families included *Erwiniaceae* (9%, 6–12.5%), *Micrococcaceae* (9%, 1.4–18.8%), *Enterobacteriaceae* (6%, 1.8–12.8%), *Enterococcaceae* (3%, 0–6.8%), and *Geodermatophilaceae* (3%, 0.8–7.3%).

**Figure 2:**
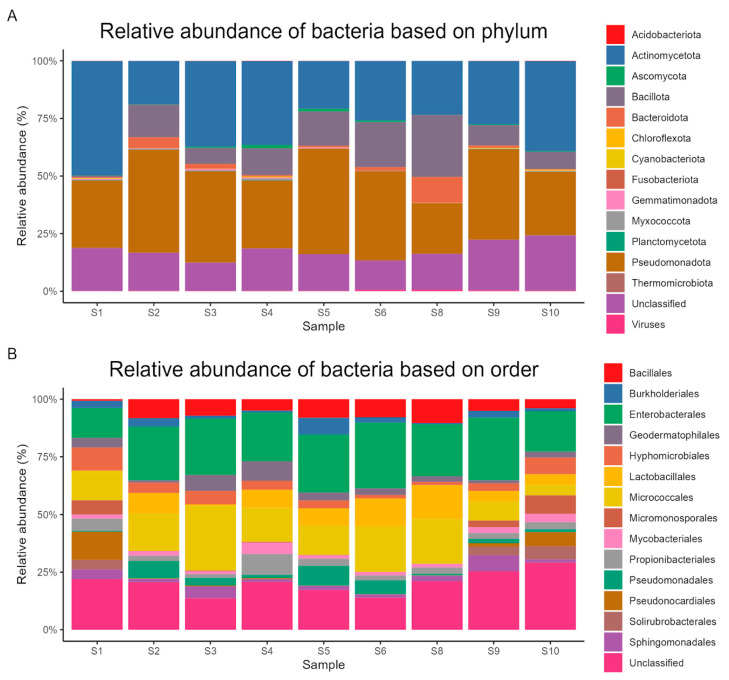
Proportional abundance of key microbial orders and phyla in samples of soil. (**A**) Relative abundance of bacteria based on phylum. (**B**) Relative abundance of bacteria based on order.

**Figure 3:**
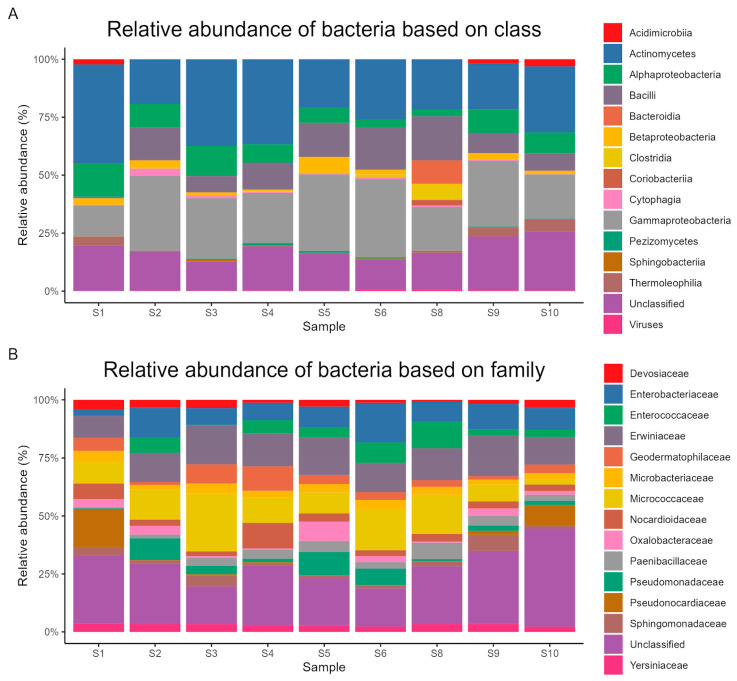
Proportional abundance of key microbial families and classes in samples of soil. (**A**) Relative abundance of bacteria based on class. (**B**) Relative abundance of bacteria based on family.

**Figure 4:**
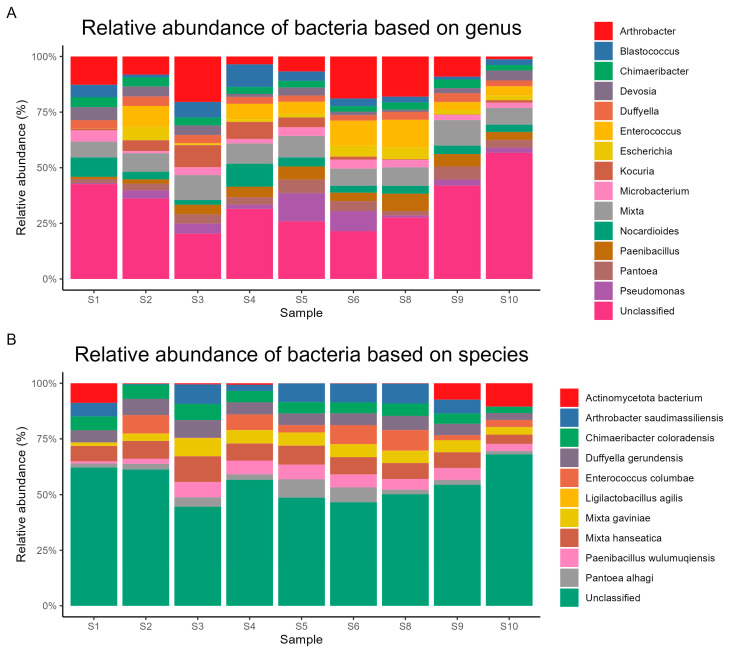
Proportional abundance of key microbial species and genera in samples of soil. (**A**) Relative abundance of bacteria based on genus. (**B**) Relative abundance of bacteria based on species.

The genera with the highest relative abundances were *Arthrobacter* (6%, 0.5–12.1%), *Mixta* (5%, 3.1–6.7%), *Enterococcus* (3%, 0–6.8%), *Nocardioides* (2%, 1.2–5.8%), and *Pseudomonas* (2%, 0.2–7.6%). The most abundant species were *Mixta hanseatica* (2%, 1.4–3.1%), *Duffyella gerundensis* (2%, 1.1–2.2%), *Arthrobacter saudimassiliensis* (2%, 0–2.7%), *Chimaeribacter coloradensis* (2%, 1–2%), and *Enterococcus columbae* (2%, 0–2.8%). Figure 2, Figure 3 and Figure 4 show the relative abundances of dominant bacterial taxa at various taxonomic levels.

### 3.3. Dominant Microbial Genera per Sample

Sample S1 was distinguished by the prevalence of *Pseudonocardia* (5%), followed by *Arthrobacter* (5%) and *Nocardioides* (4%). Sample S2 showed a lower microbial diversity, with *Enterococcus* as the most prevalent genus (4%), and *Mixta* (4%) and *Paracoccus* (4%) as the taxa with the next highest abundance. Unique microbial profiles were detected in other samples. For example, sample S3 was dominated by *Arthrobacter* (12%), followed by *Mixta* (7%) and *Kocuria* (6%). Conversely, sample S4 showed a preponderance of *Nocardioides* (6%), *Blastococcus* (6%), and *Mixta* (6%). *Pseudomonas* was the strongest frequent genus in S5 (8%), followed by *Mixta* (6%) and *Arthrobacter* (4%). Sample S6 showed a dominance of *Arthrobacter* (11%), followed by *Enterococcus* (6%) and *Pseudomonas* (5%). *Arthrobacter* was the most frequent genus in sample S8 (10%), followed by *Enterococcus* (7%) and *Bacteroides* (5%). Sample S9 was dominated by *Mixta* (6%), *Arthrobacter* (5%), and *Pantoea* (3%). Finally, sample S10 showed a predominance of *Pseudonocardia* (3%), with *Mixta* (3%) and *Actinoplanes* (3%). These results demonstrate the possible influence of several environmental conditions on the makeup of bacteria and the variety of microbial communities among the selected soil samples. The distribution of the top three genera in the selected soil samples is shown in Table 2.

### 3.4. Sample Correlation and Clustering

**Figure 5:**
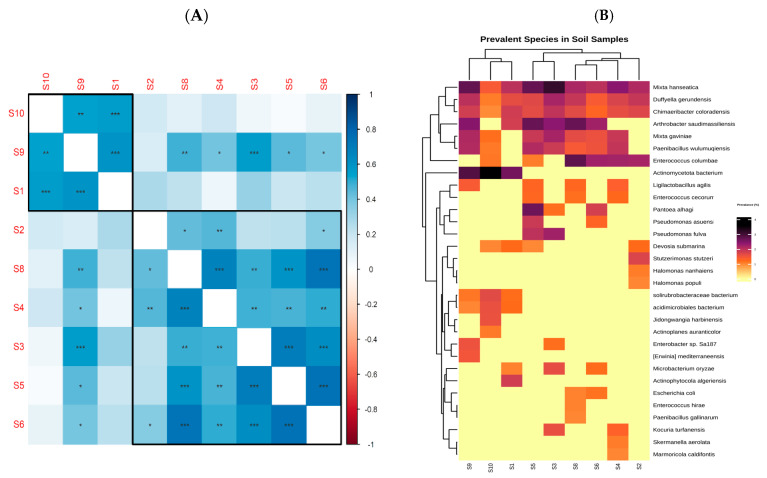
(**A**) Correlation matrix illustrating the relationships between soil samples based on bacterial species prevalence. * *p* < 0.05, ** *p* < 0.01, *** *p* < 0.001) (**B**) Heatmap and hierarchical clustering of soil samples and dominant species distribution.

Correlation and clustering analyses were performed to inspect the connections between soil samples according to the characteristics of their bacterial communities. Significant positive connections between certain samples were shown by pairwise correlation analysis. Interestingly, samples S5 and S6 showed the highest correlation (rho = 0.7, *p* = 2 × 10^−6^), suggesting that their microbial community structures were significantly comparable. Additionally, sample S8 and sample S6 exhibited a strong association (rho = 0.7, *p* = 3 × 10^−6^). Other significant correlations included S3-S5 (rho = 0.7, *p* = 1 × 10^−5^), S8-S4 (rho = 0.7, *p* = 3 × 10^−5^), S3-S6 (rho = 0.6, *p* = 2 × 10^−4^), S8-S5 (rho = 0.6, *p* = 4 × 10^−4^), S9-S1 (rho = 0.6, *p* = 5 × 10^−4^), S10-S1 (rho = 0.6, *p* = 9 × 10^−4^), and S3-S9 (rho = 0.6, *p* = 1 × 10^−3^). Based on the makeup of the microbial population, the samples were sorted into discrete groups using hierarchical clustering analysis. A cohesive cluster was established by samples S2, S3, S4, S5, S6, and S8, suggesting that their microbial populations were closely related. The clustering of samples S1, S9, and S10, on the other hand, suggested a common microbial community structure. This clustering might be due to distinct environmental factors unique to these sites, such as different soil pH, temperature variations, or specific vegetation types influencing the microbial communities. These sites may also experience unique ecological interactions or disturbances that shape their microbial profiles differently from the other samples. Figure 5A,B represent the correlation matrix and heatmap with a dendrogram, respectively, visually demonstrating the relationships between soil samples and the clustering patterns.

### 3.5. COG Functional Annotation

**Figure 6:**
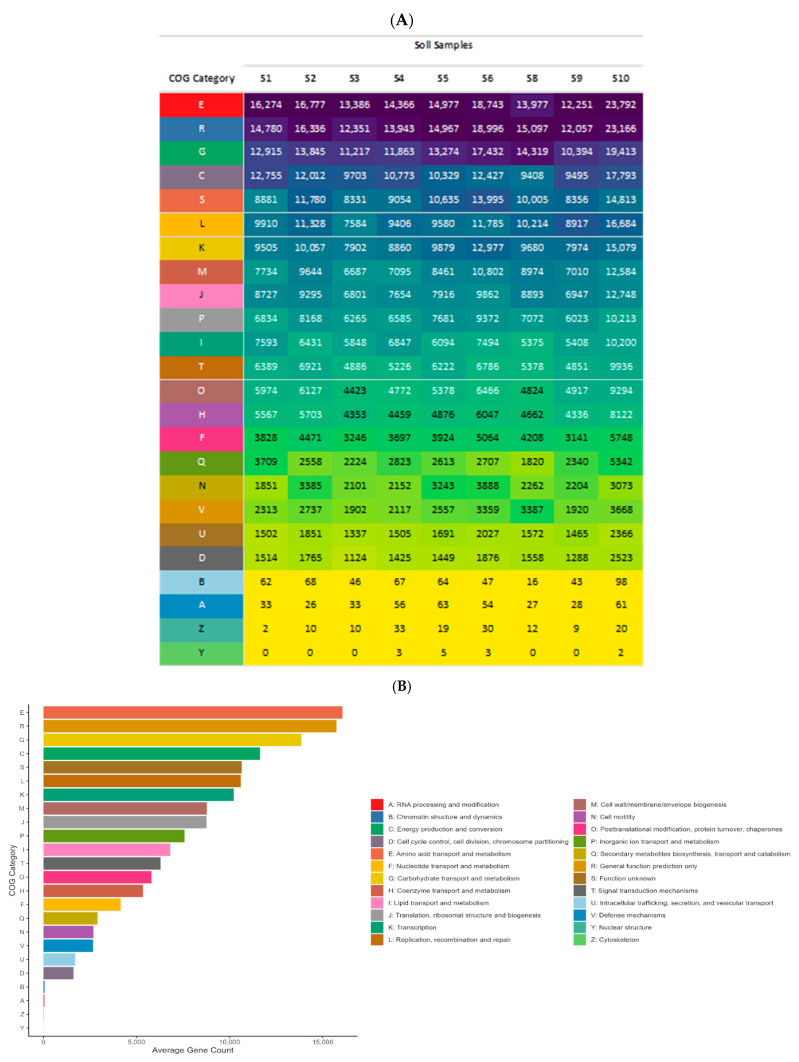
(**A**) Enriched OTUs by COG functional groups. (**B**) Average OTU counts per COG category.

COG functional annotation was carried out in order to examine the functional characteristics of the discovered protein clusters. A hierarchical classification of COG functions was established, based on the average number of clustered proteins assigned to each COG category across all samples. The following were the top ten COG categories of average protein abundance in descending order: E: Amino acid metabolism and transport (average proteins = 16,060), R: General functional prediction only (average proteins = 15,744), G: Carbohydrate metabolism and transport (average proteins = 13,852), C: Energy production and conversion (average proteins = 11,633), S: Function unknown (average proteins = 10,650), L: Replication and repair (average proteins = 10,601), K: Transcription (average proteins = 10,213), M: Cell wall/membrane/envelop biogenesis (average proteins = 8777), J: Translation (average proteins = 8760), and P: Inorganic ion transport and metabolism (average proteins = 7579). The distribution of COG categories across individual samples is shown in Figure 6A, and the enrichment of particular COG categories is shown in Figure 6B. The primary functional traits of the microbial communities residing in the soil samples are revealed by these findings. Certain COG categories may indicate adaptations to specific ecological niches or environmental conditions in certain samples.

### 3.6. Microbial Alpha Diversity

Each soil sample’s microbial diversity was described using a set of alpha diversity indices. The direct count of OTUs, known as species richness, varied significantly throughout the soil samples, ranging from 7585 to 12,007 OTUs. These results were confirmed by species richness estimators Chao1 and ACE, which indicated that samples S10, S2, and S9 had particularly high levels of diversity. The Shannon’s and Simpson’s indexes were estimated to take into consideration both species richness and evenness. Overall, sample diversity was moderate to high according to these criteria, with sample S1 exhibiting the highest diversity. Samples S10, S2, and S1 were found to have the most diverse microbial communities based on Fisher’s alpha index, another indicator of species richness, which matched the indices previously indicated. On the other hand, sample S6 consistently showed the lowest values of all the evaluated alpha diversity indices, indicating that it was the least diversified microbial community. All of these findings point to the substantial differences in microbial diversity amongst the soil samples, highlighting the impact of environmental variables on the composition of microbial communities. The values of all diversity indices employed in the analysis are shown in Table 3, which offers a thorough description of the alpha diversity found in the soil samples under investigation.

## 4. Discussion

This research uncovers new perspectives on the diversity, composition, and functional characteristics of microbial communities in the soil of selected sites within the Al-Tubaiq Reserve at KSRNR, Saudi Arabia. These insights contribute to our understanding of how microbial communities interact with their environment in this unspoiled ecosystem. To the best of our knowledge, this is the inaugural metagenomic study of the soil microbiome in the specified areas of KSRNR. Our results provide essential information for evaluating the sustainability of KSRNR ecosystems, which are influenced by both natural and human factors. Although the study is constrained by the use of a limited number of random samples across the extensive reserve, this methodology aligns with exploratory metagenomic research aimed at providing initial characterizations of microbiomes in ecologically significant but under-researched regions. Monitoring biodiversity and environmental management can be accomplished using the advanced metagenomic investigation of ambient microbial populations as an in situ sensor in the surveyed environment [23] (Garlapati et al., 2019). They can be used to signify negative and positive effects on the soil ecosystem. For instance, bacterial indicators could be used to detect changes in the ecosystem due to pollutant risks [24] (Zaghloul et al., 2020). Furthermore, some bacteria could function as biological biomarkers specific to particular habitats. For example, microbiome samples collected from deserts may contain abundant genes related to osmoregulation and dormancy and a low number of antibiotic-resistant genes, suggesting minor competition in the desert biome [25] (Nam et al., 2023). Therefore, metagenomic analysis by 16S rRNA is superior to the culture-dependent approach since it provides an accurate description and higher resolution of the microbial flora.

In this study, metagenomic sequencing analysis was used to study the diversity of ten collected soil samples from sandy soils surrounding water resources in the Tubaiq conservation area of KSRNR, Saudi Arabia. The taxonomic analysis revealed 181 phyla, 213 classes, 446 orders, 1036 families, and 4102 genera. The analysis of 16S rRNA amplicon sequencing data supported that *Pseudomonadota* was the most dominant phylum across the samples, with a relative abundance of 34%. A previous study reported that *Pseudomonadota* has the capability to face desiccation and the ability to repair DNA damage [26] (Selmani et al., 2023). In addition, *Pseudomonadota* members are copiotrophic bacteria and are able to flourish in soils rich in nutrients [27] (Han et al., 2023). Moreover, our analysis reveals the presence of the Enterococcaceae family, which is associated with contamination with enteric bacterial pathogens [28] (Fongaro et al., 2017). Our analysis also reveals the occurrence of 2% of *Enterococcus columbae*, a Gram-positive facultative anaerobic bacterium previously reported as a component of pigeon intestinal flora [29] (Baele et al., 2002). Moreover, the same percentage of *Geodermatophilaceae* was previously reported in the soil microbiome in the southwestern highlands of Saudi Arabia [30] (Yasir et al., 2015).

The presence of 30% *Actinomycetota* in soil samples is associated with the synthesis of bioactive metabolites, the decomposition of organic substances, and the biological control of soil [31] (Bhatti et al., 2017). Additionally, *Arthrobacter sp.* was the most dominant genus in the soil samples. Furthermore, a previous study reported that *Arthrobacter* strains have a significant ecological role in the interaction with host plants [32] (Fernández-González et al., 2017). Moreover, the genus *Arthrobacter* is broadly found in soils in extreme environments, such as the Cholistan desert (Pakistan) and the Xinjiang desert (China) [26,33] (Crocker et al., 2000; Selmani et al., 2023). Surprisingly, our investigation reveals the occurrence of 2% of *Arthrobacter saudimassiliensis*, which was isolated for the first time from air samples in the environment of Makkah, Saudi Arabia, in 2012 [34] (Papadioti et al., 2017).

The dominant microbial genera analysis per sample demonstrates that the bacterial communities at the sites under investigation are different. Clustering analysis shows that samples S2, S3, S4, S5, S6, and S8 are relatively close among their microbial communities. This similarity could be attributed to shared environmental conditions, such as similar soil types, moisture levels, and nutrient availability in these areas. Additionally, these sites might have comparable exposure to natural and anthropogenic factors, leading to similar microbial compositions. Samples S1, S9, and S10, on the other hand, grouped together, indicating a common microbial community pattern. This clustering might be due to distinct environmental factors unique to these sites, such as different soil pH, temperature variations, or specific vegetation types influencing the microbial communities. These sites may also experience unique ecological interactions or disturbances that shape their microbial profiles differently from the other samples. This suggests that environmental factors, including temperature and humidity, were affecting bacterial growth and genus distribution [35] (Qiu et al., 2022). However, due to the soil’s nitrogen content, the distribution of the major taxa and their proportions varied greatly between samples. Moreover, the presence of the genus *Pseudonocardia* in S1 suggests a close relationship with multiple species of fungus-growing ants, where these bacteria produce diverse secondary metabolites and protect ants from disease [36] (Goldstein and Klassen, 2020). Furthermore, the genus Nocardiodes was dominant in S4. This genus is able to endure various low-nutrient conditions and degrade pollutants in soil [37] (Ma et al., 2023). Additionally, S5 reveals an abundance of the genus Pseudomonas, which plays a key role in the biological control of soil-borne plant pathogens and the bioremediation of pollutants [38] (Garbeva et al., 2004). Soil samples S3, S6, and S8 showed a prevalence of the genus Arthrobacter, which may be due to its ability to survive under stressful conditions induced by starvation and toxic chemicals [39] (Mongodin et al., 2006).

The COG annotation shows the distribution of different enrichment patterns among the samples under investigation. Our investigation reveals that most COG annotations were involved in amino acid metabolism and transport, carbohydrate metabolism and transport, and energy production and conversion. Regulatory systems involved in amino acid metabolism have functions such as stress tolerance, biofilm development, and chemotaxis [40] (Amador et al., 2010). Moreover, carbohydrate metabolism-related genes show that the microbial flora present in these sites are able to provide energy and carbon for plant growth [41] (Cui et al., 2021).

The microbial diversity in each soil sample was assessed using a series of alpha diversity indices. The observed species richness varied significantly between samples, with counts varying from 7585 to 12,007 OTUs. The species richness estimators Chao1 and ACE supported our findings, showing that samples S10, S2, and S9 exhibited very high levels of variety. S8, S9, and S10 are from water points constructed to support reintroduced wildlife with essential water in the Tubaiq conservation area in KSRNR, especially during droughts, and the presence of wild ungulate species and their grazing activities, which is considered a light level of grazing given the area and number of ungulates in these areas. According to Zhang et al. (2023) [42], soil bacterial diversity was higher under light grazing treatment compared to heavy grazing. Therefore, the influence of wildlife on soil bacterial community diversity warrants further exploration in the context of ecological dynamics. Additionally, a previous metagenomic study by Fierer et al. (2012) [43] reported significant positive linear correlations between the richness of functional genes and bacterial species diversity. The current study shows that the detected microbial community includes three *Pseudomonadota*/*Proteobacteria* classes: *Gammaproteobacteria* 24%, *Alphaproteobacteria* 8%, and *Betaproteobacteria* 2%. *Alphaproteobacteria* and *Gammaproteobacteria* are commonly found in freshwater systems, particularly in areas with significant human activity, and play crucial roles in degrading organic matter and cycling nutrients [44] (Małecka-Adamowicz and Kubera 2021). *Gammaproteobacteria* is the most genus-rich taxon of the Prokaryotes, containing about 250 genera [45] (Garrity et al., 2005). It is a class of several ecologically, scientifically, and medically important groups of bacteria. Ecologically, Gammaproteobacteria serve as potential biomarkers for pollution [46] (Ghai 2011) and have significant applications in various fields. They are utilized in degrading pollutants and toxins in contaminated areas contributing to environmental remediation [47] (Kasai 2001) and the purification of some industrial wastewater by degrading organic matter and reducing pollutants [48] (Kobayashi et al., 1973). Future studies could focus on quantifying specific pollutants and correlating their levels with the abundance of Gammaproteobacteria to further validate this relationship. In agriculture, they play a vital role in nitrogen fixation, leading to the enhancement of soil fertility and, hence, plant growth [49] (Baker et al., 2015). In addition, they play a role in energy production through microbial fuel cells [50] (Qian and Morse 2011). Medically, species such as Escherichia coli play a vital role in biotechnology by producing antibiotics, enzymes, and various other bioactive compounds [51] (Williams et al., 2010).

*Hyphomicrobiales* is an order within the class *Alphaproteobacteria* that belongs to the phylum *Pseudomonadota/Proteobacteria*, comprising various families, such as *Rhizobiaceae*, *Brucellaceae*, and *Methylocystaceae*. These families play crucial ecological roles, including nitrogen fixation, organic matter decomposition, and nutrient cycling [52] (Garrity 2007). Members of *Hyphomicrobiales* also form symbiotic relationships with plants, promoting growth and protecting against pathogens [53] (Sprent and Platzmann, 2001). Their genomic diversity, which includes prophages and adaptive mechanisms, showcases their evolutionary resilience [52] (Garrity 2007). However, many species within this order remain understudied, presenting opportunities for future research, particularly with the advancements in metagenomics and high-throughput sequencing techniques [54] (Rohwer and Edwards, 2002). Understanding Hyphomicrobiales’ roles and potential applications could significantly enhance agricultural productivity and environmental sustainability. Enterobacterales include a diverse group of Gram-negative bacteria within the phylum Pseudomonadota, known for their crucial roles in environmental and clinical fields. This order encompasses well-known genera, such as *Escherichia, Salmonella, Klebsiella, Enterobacter, Shigella, and Proteus*. These bacteria participate in various ecological processes, including nutrient cycling and plant–microbe interactions while also being prominent pathogens for humans and animals [55] (Janda and Abbott, 2021). Significant heterogeneity in the diversity and composition of sediment bacteria was recently discovered in the Tabuk region by Al-Awthan et al. (2024) [56]. The research demonstrated how important microbial diversity is to ecological balance and environmental-influenced biogeochemical processes. So, these results offer crucial information for the KSRNR’s water resource conservation and sustainable management. While 16S rRNA sequencing is a powerful tool for microbial community analysis, it may not always provide definitive species-level identification (65 to 83% species identification). This is due to factors like limited sequence data in databases, overlapping sequences between species, and the existence of novel taxa (Janda et al., 2007). However, in our study, we were primarily interested in understanding the overall microbial community structure and diversity, including finer-scale details like species-level information. Although not always precise, this level of analysis provides valuable insights into the community’s complexity. Therefore, we included species-level information in our study, acknowledging its limitations but recognizing its contribution to our overall understanding.

## 5. Conclusions

The microbial communities of soil around the lakes in KSRNR provide the basis of the biogeochemical cycles and food chain. The purpose of this study was to evaluate the microbial community alterations in the soil at manmade water points and natural lakes in the KSRNR. Using 16S rRNA metagenomics, we identified soil bacterial diversity and composition at 10 locations surrounding water resources in the Tubaiq conservation area. Our objective was to compile a thorough database of microbial communities and their richness while connecting it to human activity in the vicinity of water resources.

Our findings suggest that changes in the composition of the microbial communities at the sites under investigation are probably the result of human activity. These findings enhance our understanding of factors influencing the soil microbiome at the natural lakes and artificial water points in the KSRNR. This knowledge is crucial for developing environmental management strategies and planning future restoration efforts for the affected ecosystems. Our study provides a valuable foundation for future research and conservation initiatives aimed at preserving the ecological integrity of these vital habitats.

## Figures and Tables

**Table 1:** Physiographic features of the study locations and soil samples.

Samples	Type	Latitude (°)	Longitude (°)	Elevation (M)	Humidity (%)	Temperature (°C)
S1	Sandy	28.5	37.5	917 m	30	27
S2	Sandy	28.5	37.6	879 m	32	27
S3	Sandy	28.7	37.8	839 m	26	33
S4	Sandy	29.0	37.9	790 m	19	37
S5	Sandy	29.3	37.7	756 m	29	29
S6	Sandy	29.3	37.6	764 m	25	30
S8	Sandy	29.5	37.6	871 m	20	32
S9	Sandy	29.4	37.4	919 m	18	34
S10	Sandy	29.4	37.5	876 m	19	33

**Table 2:** The distribution of the top three genera in the selected soil samples.

Genus	Soil Samples								
	S1	S2	S3	S4	S5	S6	S8	S9	S10
*Pseudonocardia*	5%	0%	0%	0%	0%	0%	0%	0%	3%
*Mixta*	0%	4%	7%	5%	6%	0%	0%	6%	3%
*Actinoplanes*	0%	0%	0%	0%	0%	0%	0%	0%	3%
*Arthrobacter*	5%	0%	12%	0%	4%	11%	10%	5%	0%
*Nocardioides*	4%	0%	0%	6%	0%	0%	0%	0%	0%
*Enterococcus*	0%	4%	0%	0%	0%	6%	7%	0%	0%
*Paracoccus*	0%	4%	0%	0%	0%	0%	0%	0%	0%
*Kocuria*	0%	0%	6%	0%	0%	0%	0%	0%	0%
*Blastococcus*	0%	0%	0%	6%	0%	0%	0%	0%	0%
*Pseudomonas*	0%	0%	0%	0%	8%	5%	0%	0%	0%
*Bacteroides*	0%	0%	0%	0%	0%	0%	5%	0%	0%
*Pantoea*	0%	0%	0%	0%	0%	0%	0%	3%	0%

**Table 3:** Alpha diversity metrics for every sample of soil.

Sample ID	Observed	Chao1	ACE	Shannon	Simpson	Fisher
S1	9382.00	14,693.65	14,835.37	6.50	0.99	2181.99
S2	10,324.00	17,948.56	18,556.59	6.26	0.99	2472.35
S3	7585.00	12,906.87	13,508.58	5.88	0.99	1782.19
S4	8450.00	14,842.46	15,565.87	6.03	0.99	1959.79
S5	7616.00	14,434.60	15,073.10	5.53	0.98	1716.04
S6	7778.00	14,145.91	14,771.65	5.58	0.99	1704.41
S8	8090.00	14,283.78	14,945.92	5.79	0.99	1872.87
S9	8545.00	15,235.13	15,641.04	5.80	0.98	2099.33
S10	12,007.00	19,403.97	19,696.49	6.26	0.99	2677.59

## Data Availability

The original contributions presented in this study are included in the article. Further inquiries can be directed to the corresponding author.
